# Digital Papillary Adenocarcinoma: The Detection of Low-Risk Human Papillomaviruses and the *BRAF* p.V600E Mutation in a Subset of Cases

**DOI:** 10.3390/dermatopathology11030018

**Published:** 2024-06-28

**Authors:** Feifan Chen, Priyadharsini Nagarajan, Phyu P. Aung

**Affiliations:** Department of Anatomic Pathology, MD Anderson Cancer Center, The University of Texas, Houston, TX 77030, USA; fchen3@mdanderson.org

**Keywords:** digital papillary adenocarcinoma, HPV 42, LR-HPV, *BRAF* p.V600E

## Abstract

Digital papillary adenocarcinoma (DPA) is a rare malignant neoplasm which arises from the sweat glands and has metastatic potential. DPA exhibits a wide range of architectural features and exhibits low-grade to high-grade features, so distinguishing DPA from benign skin neoplasms, including acral hidradenoma, poses significant diagnostic challenges. The recent literature suggests a strong association between DPA and human papillomavirus (HPV) 42, a low-risk HPV (LR-HPV) subtype, and a possible association between DPA and *BRAF* p.V600E. To explore these associations, we assessed the utility of in situ hybridization (ISH) for LR-HPV (types 6, 11, 40, 42, 43, 44) and immunohistochemistry (IHC) for *BRAF* p.V600E in diagnosing DPA and distinguishing DPA from acral hidradenoma. With institutional review board approval, we retrospectively identified 15 specimens of DPA (from 13 patients) and 3 cases of acral hidradenoma. Of the 13 DPA cases, 6 were negative for LR-HPV and *BRAF* p.V600E; 6 were positive for only LR-HPV; and 1 was positive for only *BRAF* p.V600E but negative for LR-HPV. All three cases of acral hidradenoma were negative for LR-HPV and *BRAF* p.V600E. As our sample size is limited, larger studies are needed to assess the value of detecting LR-HPV and *BRAF* p.V600E in the distinction of DPA and acral hidradenoma. However, our findings indicate a stronger association of DPA with LR-HPV than with *BRAF* p.V600E.

## 1. Introduction

Digital papillary adenocarcinoma (DPA) is an exceedingly rare malignant neoplasm arising from the sweat glands. It commonly presents as a slow-growing and deep-seated lesion and predominantly affects the hands and feet of white men in their fifth to sixth decades [1,2,3,4,5]. DPA exhibits a tendency for local recurrence (rate of 5% to 21%) and metastasis (26% to 50%) to the lymph nodes and lungs, necessitating amputation [1,2,3,4].

Histologically, DPA manifests as a multinodular lesion within the dermis and/or subcutaneous tissue without overlying epidermal connection. It exhibits a wide range of architectural patterns, including solid, cystic, papillary, pseudopapillary, and glandular, either in combination or with a predominance of one or two patterns. Furthermore, DPA exhibits a range of low-grade to high-grade features, including cytologic atypia, necrosis, mitotic activity, and infiltrative growth patterns [1,2,3,4]. Due to its various histomorphologic features that sometimes overlap with other (benign) entities, DPA has historically been a diagnostic challenge, as reflected in the numerous names given to this entity over the years. DPA was first described by Helwig in 1979 and termed “aggressive digital papillary adenoma” [6]. In a subsequent study at the Armed Forces Institute of Pathology, Kao et al. subclassified the tumors as “aggressive papillary digital adenoma” and “aggressive papillary digital adenocarcinoma”, based on their belief that the neoplasm had benign and malignant subtypes indicated by low-grade and high-grade features, respectively [1]. However, in a retrospective study at the same institution, published in 2000, Duke et al. found that several cases initially diagnosed as “aggressive papillary digital adenoma” recurred locally and a few such cases metastasized [2], suggesting that even tumors formerly termed “adenoma” or with low-grade features should be considered malignant and be termed “adenocarcinoma”.

Therefore, it is of upmost importance to differentiate DPA from benign mimickers, the most common of which is acral hidradenoma. To differentiate the two, a panel of immunohistochemical (IHC) studies have been suggested to identify luminal and myoepithelial cells [3,7]. Recent studies have shown a strong association between DPA and human papillomavirus 42 (HPV 42), a low-risk HPV (LR-HPV) subtype, by molecular studies and have demonstrated diffuse positivity for HPV 42 by in situ hybridization (ISH) in cases of DPA [8,9,10,11,12,13,14]. Other studies showed that a subset of DPA cases were associated with *BRAF* mutations [14,15,16]. The aim of our study was to evaluate the utility of ISH for LR-HPV and IHC for *BRAF* p.V600E in the diagnosis of DPA and to explore the association of DPA with LR-HPV and *BRAF* p.V600E.

## 2. Materials and Methods

After receiving approval for this study from the Institutional Review Board of The University of Texas MD Anderson Cancer Center (approval number 2020-0658), we searched the institutional archives to identify cases of DPA or acral hidradenoma diagnosed during 2011–2023. Cases with readily available paraffin-embedded blocks or at least 3 unstained recut slides were chosen. For each case, 1 slide was stained with hematoxylin–eosin for the evaluation of tissue adequacy, tumor burden, and diagnosis. Ultimately, 15 specimens of DPA, from 13 patients, and 3 cases of acral hidradenoma were selected.

ISH for LR-HPV, which includes HPV subtypes 6, 11, 40, 42, 43, and 44, was performed by the RNAScope HPV LR6 assay, Advanced Cell Diagnostics/Leica Biosystems catalog 407608, and IHC for *BRAF* p.V600E was performed with the VE1 monoclonal antibody, Ventana catalog 790-5095 at our CLIA-certified clinical laboratory using appropriate mRNA control slides. All findings on hematoxylin–eosin staining, ISH, and IHC were reviewed by 2 authors (P.P.A. and F.C.).

## 3. Results

The demographic, clinical, and pathologic findings of the cases are summarized in Table 1.

### 3.1. DPA Cases

**Figure 1:**
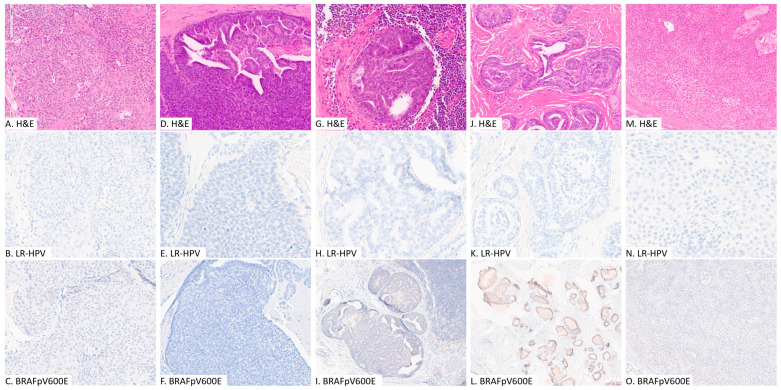
Representative images of digital papillary adenocarcinoma (**A**–**L**) and acral hidradenoma (**M**–**O**); hematoxylin and eosin stain (H&E), immunohistochemical study for BRAFpV600E and in situ hybridization analysis for low-risk human papilloma viruses. (**A**–**C**): patient #3, WT group, primary tumor; (**D**–**F**): patient #7, LR-HPV group, primary tumor; (**G**–**I**): patient #9, LR-HPV group, lymph node metastasis; (**J**–**L**): patient #13, BRAF group, primary tumor; (**M**–**O**): patient #15, acral hidradenoma.

Based on the IHC and ISH results, of the 13 cases of DPA, 6 were negative for both LR-HPV and *BRAF* p.V600E (WT group), 6 were positive for only LR-HPV (LR-HPV group), and 1 was positive for only *BRAF* p.V600E (BRAF group) (Table 1 and Figure 1).

**Table 1:** Demographic, clinical, and pathologic findings of specimens from patients with DPA and acral hidradenoma.

Group	Pt	Age, y	Sex	Race and Ethnicity	Anatomic Site	Tumor Size, mm	Metastasis	Follow-up, y	LR-HPV by ISH	*BRAF* p.V600E by IHC	Management
	**Digital papillary adenocarcinoma**										
WT	1	48	F	White	Left ankle, medial side	4	None	6	Negative	Negative	Wide local excision
WT	2	41	M	White	Right index finger	1.5	SLN (2/4) ^a^; lung	13	Negative	Negative	Amputation of digit; SBRT of lung metastasis
WT	3	39	F	White	Right heel	28	SLN (1/5) ^a^	11	Negative	Negative	Wide local excision
WT	4	54	M	White	Right middle finger	3.6	None	3 (deceased, no record)	Negative	Negative	Ray amputation
WT	5	23	F	Unknown	Right second toe, distal plantar surface	Unknown	None	Unknown	Negative	Negative	Unknown
WT	6	34	F	African	Right ring finger, palmar surface	Unknown	None	2	Negative	Negative	Partial amputation
LR-HPV	7	64	M	White	Left middle finger	10	None	3	Positive	Negative	Partial amputation
LR-HPV	8	64	M	White	Right middle finger	45	None	2	Positive	Negative	Partial amputation
LR-HPV	9	53	M	White	Left middle finger	27	SLN (1/2) ^a^; LN (2/20) ^a^	Unknown	Positive	Negative	Distal amputation
LR-HPV	10	62	F	White	Right middle finger	12	None	3	Positive	Negative	Ray amputation
LR-HPV	11	51	M	Unknown	Left long finger	Unknown	None	Unknown	Positive	Negative	Unknown
LR-HPV	12	49	M	White	Right finger	Unknown	LN (3/24) ^a^; lung	4	Positive	Negative	Amputation; chemotherapy for metastasis
BRAF	13	42	F	Unknown	Left middle finger	6.5	None	Unknown	Negative	Positive	Unknown
	**Acral hidradenoma**										
	14	67	F	White	Left foot, plantar surface	3	None	7	Negative	Negative	Excisional biopsy
	15	44	M	Latino	Left wrist, dorsal surface	8	None	3	Negative	Negative	Excision
	16	76	M	White	Right foot, dorsal surface	5	None	3	Negative	Negative	Excision

F, female; LN, lymph node; M, male; Pt, patient; SLN, sentinel lymph node; WT, wild-type. ^a^ Ratios indicate (number of nodes with metastases)/(total number of nodes examined).

For the WT group, the mean age was 40 years (range 23 to 54), and there was a female predominance (M:F; 1:2). The average tumor size was 9.3 mm (range 1.5 to 28 mm), distributed equally among the upper and lower extremities. Sites of metastasis included the sentinel lymph nodes in two cases, one of whom also developed metastasis to the lung.

For the LR-HPV group, the mean age was 57 years (range 49 to 64), and there was a male predominance (M:F; 5:1). All the tumors involved the fingers. The mean tumor size was 23.5 mm (range 10 to 45 mm). Two patients had metastasis to lymph nodes, and one had pulmonary metastasis as well. LR-HPV positivity was noted solely in the tumor cells in both primary and metastatic tumors. The ISH showed variable intensity, from weak to strong.

For the BRAF group, the single case was a 6.5 mm lesion on the left middle finger of a 42-year-old female.

Overall, the 13 patients with DPA had an average age of 46 years (range 39 to 53) at presentation with a male predominance (M:F; 3:1). The average tumor size was 18.9 mm (range 1.5 to 28 mm).

The lesion positive for only *BRAF* p.V600E was received as a consultation, and minimal clinical history was provided. Histomorphologic examination showed an epithelial neoplasm involving the dermis exhibiting glandular differentiation with enlarged cystic spaces and focal papillary features with low-grade cytologic atypia and focal stromal invasion in the periphery.

In contrast, the cases negative for and LR-HPV and *BRAF* p.V600E and the cases positive for only LR-HPV exhibited a range of histomorphologic features that included papillary, solid, cystic, and glandular architectural patterns, low-grade and high-grade cytologic atypia, and invasive patterns of growth. These patterns were also reflected in the four cases with metastatic disease. Metastasis was more common in DPA arising from the fingers, compared to the lower extremities.

### 3.2. Acral Hidradenoma Cases

The three acral hidradenomas were negative for both LR-HPV and *BRAF* p.V600E. The mean age at diagnosis was 62 years (range 44 to 76). Two were located on the foot, and one on the wrist. The mean tumor size was 5 mm (range 3 to 8 mm) (Figure 1).

## 4. Discussion

In this study of the utility of ISH for LR-HPV and IHC for *BRAF* p.V600E in the diagnosis of DPA, we found that 6 of the 13 cases were associated with LR-HPV only, 1 was associated with *BRAF* p.V600E only, and the rest were not associated with either LR-HPV or *BRAF* p.V600E. DPA appears to be more strongly associated with LR-HPV and less strongly associated with *BRAF* p.V600E. Additionally, our three cases of acral hidradenoma were negative for both LR-HPV by ISH and *BRAF* p.V600E by IHC.

Our cases that were associated with LR-HPV showed positive hybridization signals exclusively in the tumor cells in both primary and metastatic tumors. The intensity varied from weak to strong. While the exact cause is unclear, a variable combination of the following factors may be attributable: a low concentration of HPV 42 probe in the LR-HPV ISH panel compared to other LR-HPV subtypes and variable copy numbers of HPV 42 virus integrated in the tumor genomic DNA. A comparative study of HPV 42 ISH and LR-HPV ISH may help further confirm the diagnostic applicability of LR-HPV ISH. Currently, no other DPA-mimicking lesions are known to be associated with HPV 42 or other LR-HPV types (types 6, 11, 40, 42, 43, and 44); however, a study on the expression of LR-HPV by ISH in other eccrine neoplasms may help determine the specificity. Additionally, further studies comparing the detection of LR-HPV using ISH versus polymerase chain reaction (PCR) could help assessing the value of ISH as a diagnostic tool.

For the sole case associated with *BRAF* p.V600E by IHC, we acknowledge that this may represent a tubular adenoma, given the recent reports in the literature; however, due to our small sample size and the still-controversial literature, we believe that more studies with larger sample sizes are required [15,16,17].

In conclusion, our study supports previous studies suggesting an association between DPA and LR-HPV and possibly *BRAF* p.V600E [8,9,10,11,12,13,14,15,16]. Notably, LR-HPV positivity by ISH may aid in the diagnosis of DPA, but the LR-HPV findings should be interpreted alongside other clinical and histological findings and should not be used as the sole deciding factor. Conversely, the absence of LR-HPV expression by ISH is nonspecific and does not rule out the diagnosis of DPA.

## Data Availability

Data are contained within the article. The original contributions presented in the study are included in the article; further inquiries can be directed to the corresponding author.
